# Comprehensive assessment of harmful heavy metals in contaminated soil in order to score pollution level

**DOI:** 10.1038/s41598-022-07602-9

**Published:** 2022-03-03

**Authors:** Haodong Zhao, Yan Wu, Xiping Lan, Yuhong Yang, Xiaonan Wu, Liyu Du

**Affiliations:** 1 College of Land and Environment, Shenyang Agricultural University, Shenyang, Liaoning China; 2 Northeast Key Laboratory of Conservation and Improvement of Cultivated Land (Shenyang), Ministry of Agriculture, P.R. China National Engineering Laboratory for Efficient Utilization of Soil and Fertilizer, Shenyang, Liaoning China; 3 Rural Energy and Environmental Protection Department, Liaoning Agricultural Development Center, Shenyang, Liaoning China; 4 College of Bioscience and Biotechnology, Shenyang Agricultural University, Shenyang, Liaoning China

**Keywords:** Environmental sciences, Solid Earth sciences

## Abstract

Soil-related problems have grown up to be a major threat to human society. Scientific evaluation is helpful to understand the status of soil pollution and provide reference to further work. In this situation, Liaoning Province, a typical industrial and agricultural province in Northeast China, was selected as a case study region. It reviewed 200 studies published between 2010 and 2020 and recorded related data of soil heavy metal. It used model method and index method to evaluate the agricultural region. The comprehensive assessment score of Liaoning pollution level was 0.8998. Dalian was 0.9536, ranking first among the 14 cities. Huludao and Jinzhou were 0.7594 respectively, ranked the last. Heavy metal accumulation in different cities stemmed from different sources, including weathering of parent materials, industrial wastes, sewage irrigation, and mining activities. In general, the pollution level of heavy metal in Liaoning was at low risk level, but it still needs to pay attention to the health risk of heavy metal and the input of heavy metal into the soil, especially cadmium (Cd). This study provides a comprehensive assessment of soil heavy metal pollution in Liaoning, while identifying policy recommendations for pollution mitigation and environmental management.

## Introduction

Heavy metals are common pollutants in the soil environment, namely arsenic (As), cadmium (Cd), chromium (Cr), mercury (Hg), lead (Pb), copper (Cu), zinc (Zn), nickel (Ni). This type of contamination is biologically toxic, widely distributed, and persists long-term in soil environment^1^. With the rapid development of economy and society, a variety of heavy metals contaminated soil threatens the environment and public health^2^. In China, soil in the agricultural regions has been partly contaminated by heavy metal, which lead to the decreasing availability of farmland^3^. The over standard rate of soil pollution is 16.1%, among which the Cd, As, Hg, Pb, Cr over standard rates of heavy metals are as high as 7.00%, 2.70%, 1.60%, 1.50%, 1.10%, respectively^4^. In 2009, the global emission of heavy metal Cd reached 743.77 tons^5^. Over the past 50 years, around 30,000 tons of Cr and 800,000 tons of Pb have been released into the environment around the world^6^. Most of these heavy metals have already been accumulated in the soil. However, chronic exposure to heavy metal has harmful consequences for human beings like lung cancer, bone fractures^7,8^. Under the circumstance, remediation techniques of contaminated soil and pollution assessment have been widely concerned both domestically and globally.

A lot of studies have been conducted on remediation techniques for heavy metal polluted soil, including in-situ remediation techniques (surface capping, encapsulation, electrokinetic extraction, soil flushing, chemical immobilization, phytoremediation, bioremediation) and ex-situ remediation techniques (landfilling, soil washing, solidification, vitrification)^9,10^. These remediation techniques focus on reducing the maximum or bioavailable concentration of heavy metal in the soil^11,12^. Although these methods have high performance, most of them are expensive, harmful to the environment and time-consuming^13,14^. These traditional methods to remediate polluted soil have many limitations and a certain degree of threat. Therefore, it is essential to deploy scientific pollution assessment and advanced remediation technologies that can effectively and safely remediate heavy metal polluted soil^15^.

Heavy metal pollution assessment of farmland soil is great significance to control and mitigate the increasingly severe soil heavy metal pollution. It is the foundation and premise to reduce the risk of heavy metal pollution. The commonly used methods of assessing heavy metal pollution of soil locally and internationally can be roughly divided into the index and model methods. The index methods include single pollution, pollution load, and cumulative indexes, etc. The model methods include the enrichment factor method and potential ecological hazard index method, etc.^16^. At present, there is no unified standard for the evaluation of soil environmental quality at home and abroad^17^. On the one hand, the existing evaluation methods have their own advantages and disadvantages^18,19^. As the pollution range contained in the function is too narrow, grey clustering will appear unreasonable phenomenon^20^. Nemero index overemphasizes the influence of the highest score index^21^. On the other hand, the studies mainly focus on the key areas, such as sewage irrigation area, mine area^22^. And most studies only focus on one specific heavy metal, such as Hg, As and Cd^23^. Furthermore, various stakeholders such as regulatory authorities, landowners, and academia may have different interests and views on the effectiveness of remediation^24^. These limitations seriously affect our overall knowledge on heavy metal pollution level. Therefore, more scientific, more comprehensive, and more reliable macro-evaluation method is a top priority need.

For fill this gap, this study determined the concentrations of heavy metal in 710 soil samples from 14 cities in Liaoning agricultural sites. These soil samples are a collection of data from other published works. This study defined the weight coefficients of 8 heavy metals and 4 soil quality indexes. It used geostatistics and fuzzy mathematics to analyse harmful heavy metals in soil to further understand the impact of human activities on the quality of farmland soil. On the basis, the heavy metal pollution level and associated risk were evaluated. The objectives were as follows: (1) To analyze the heavy metal accumulation in different cities. (2) To evaluate potential ecological risk of heavy metal in soil on the city level. (3) To determine the health risk caused by heavy metal in three exposure pathways. (4) To analyze the relationship of 8 heavy metals through principal component analysis (PCA) and correlation analysis (CA). (5) To establish a score approach based on soil quality indexes to comprehensively assessment contaminated soil pollution level. It will enhance our knowledge of the comprehensive soil heavy metal pollution status across Liaoning and provide valuable information for soil management, soil remediation and soil contamination control.

## Materials and methods

### Data collection and processing

Liaoning is the core area of the northeast old industrial base^25^. This study reviewed a series of studies on soil heavy metals in agricultural regions of Liaoning published between 2010 and 2020. The main literature databases including Web of Science, China National Knowledge Infrastructure (CNKI). The sampling processing methods used in the selected literature have been widely accepted by the scientific community. The studies used should meet the following regulations: (1) The soil samples should be collected from the soil at depth within 20 cm. 3–5 soil samples were taken at each sampling site, and then mixed thoroughly to give a composite sample^26^. (2) Soil samples treatment and chemical analysis process of the selected studies should comply with the requirements listed in the HJ/T166-2004^27^. This study collected 8 heavy metal concentrations of 710 sampling sites in agricultural regions from 200 studies. And the sample numbers in different cities are presented in Table 1.

**Table 1:** Number of samples and pH value of 14 cities in Liaoning province.

City	Number of points	pH < 5.50	5.50 ≤ pH < 6.50	6.50 ≤ pH < 7.50	pH ≥ 7.50
Shenyang	99	45	22	22	10
Dalian	30	8	10	10	2
Anshan	71	20	17	25	9
Fushun	29	16	10	1	2
Benxi	22	13	5	3	1
Dandong	36	19	11	4	2
Jinzhou	84	34	20	15	15
Yingkou	35	6	13	13	3
Fuxin	48	17	11	9	11
Liaoyang	30	13	13	4	0
Panjin	22	1	3	6	12
Tieling	63	20	18	17	8
Chaoyang	59	7	13	15	24
Huludao	82	46	23	12	1
Total	710	265	189	156	100

### Single factor assessment

Single factor index is the simplest soil environmental quality index^28,29^. Heavy metal concentrations were compared to standards (GB15618-2018)^30^. In the calculation process, it is important to exclude outliers from the data matrix according to statistical methods. See supplementary materials for boxplot method.

The specific calculation formula is as follows:

1 $$ m = C_{i}/C_{n} $$

where m is a single factor index, C_i_ is the measured value of ground i term factor, C_n_ is the standard value of the i-th factor.

### Geological accumulation index assessment

The geological accumulation index (I_geo_) was proposed by Müller^31^ and has been widely used in heavy metal studies^32^. The enrichment of heavy metals in the soil can be determined by comparing the current values with the background values. The I_geo_ of the tested soil was calculated using the following equation:

2 $$ I_{geo} = \log_{2} \left( {Cn/1.5B_{n} } \right) $$

where C_n_ is the measured concentration of every heavy metal found in farmland soil (mg/kg), and B_n_ is the geological chemical background value (Table S1) of the heavy metals found in soil (mg/kg). See supplementary materials for other details (Table S2).

### Ecological risk index assessment

The potential ecological risk index (ER) was used to determinate the potential ecological risk of heavy metal accumulation in soil^33^. Soil heavy metal potential ecological risk assessment is carried out soil heavy metal properties and environmental behavior. It can also take into account the synergistic effects of various elements, pollution level, and environmental associations with heavy metal. The integrated potential ecological risk index (RI) combines the value of ER of each heavy metal. The specific calculation formula is as follows:

3 $$ CF = Cn\left( {sample} \right)/Cn\left( {Crust} \right) $$

4 $$ ER = Tr \times CF $$

5 $$ RI = \sum {Er} $$

where Tr is the toxicity coefficient and CF is the pollution factor. See supplementary materials for other details (Tables S3 and S4).

### Health risk assessment

Human health risk assessment is the process of assessing the nature and possibility of adverse health effects in humans who may be exposed to chemicals in polluted environmental media. Potential non-carcinogenic health risk and potential carcinogenic health risk (PCR) can be estimated by target hazard quotients (HQ) and the hazard index (HI)^34–36^. HI > 1 implies potential non-carcinogenic risk. If the total potential carcinogenic health risk (TCR) is greater than 1 × 10^–4^, it indicates a high carcinogenic risk to human body. If the TCR is less than 1 × 10^–6^, it means the potential carcinogenic health caused by heavy metal exposure can be negligible. There are three exposure routes (ingestion, dermal absorption, and breathing inhalation) to evaluate the health risk of heavy metal in soil. See supplementary materials for additional details.

### Comprehensive assessment

The comprehensive assessment was divided according to three steps: index determination, weight analysis, and quantitative analysis. The sub-objectives of comprehensive assessment were split into the following categories: single factor assessment (SF), geo-accumulation assessment (GEO), potential ecological risk assessment (PER) and human health risk assessment (HHR). The four indexes were combined in a more comprehensive index to analyze the heavy metal pollution level of farmland soil. Analytic hierarchy process (AHP) and Delphi method were used to determine the membership degree of the evaluation indexes. In the comprehensive assessment, the core idea is to quantify evaluation index and transform it into a quantitative value^37^. The comprehensive assessment score value ranged from 0.00 to 1.00. The higher the score, the lower the pollution level was. The scoring basis was obtained from current national, industry standard, or related theoretical studies.

There are 20 scientists from soil science, heavy metal pollution, environmental impact assessment, and other fields. They were invited for the questionnaire survey which aimed to determine the importance of matrices among the four methods. The importance of assessment methods was compared using Saaty's scale of 1–9 (Table S5) and a comparative judgment matrix was constructed (Table S6). Finally, surveys were summarized to calculate the matrices and the final weight through a consistency test. The steps of the summary method were in supplement material. The weight coefficient specific calculation formulas are presented in Eq. (6) and Eq. (7). The comprehensive assessment calculation formulas are shown in Eq. (8) and Eq. (9). This assessment method can digitize the pollution level of the heavy metal and directly assess pollution status by using a score.

6 $$ \sum\limits_{i = 1}^{n} {Wi = 1} $$

7 $$ Wi = Ti/\sum\limits_{i = 1}^{n} {Ti} $$

8 $$ T = \sum\limits_{i = 1}^{n} {Mi \times Wi} $$

9 $$ Mi = \sum\limits_{i}^{m} {Si \times Ws} $$

T_i_ represents the toxic coefficients of one certain heavy metal. T represents the total score of the comprehensive assessment result. M_i_ represents the score of one certain evaluation method. S_i_ represents the score of one certain heavy metal. W_i_ represents the weight of one certain assessment method, and W_s_ represents the weight of one certain heavy metal.

### Ethical approval and consent to participate

Written informed consent for publication of this paper was obtained from the Shenyang Agricultural University of Land and Environment and all authors.

### Consent to publication

Written informed consent was obtained from the authors for publication of this report and any accompanying images. A copy of the written consent is available for review by the Editor-in-Chief of this journal.

## Results and discussion

### Distribution of examined sites

**Figure 1:**
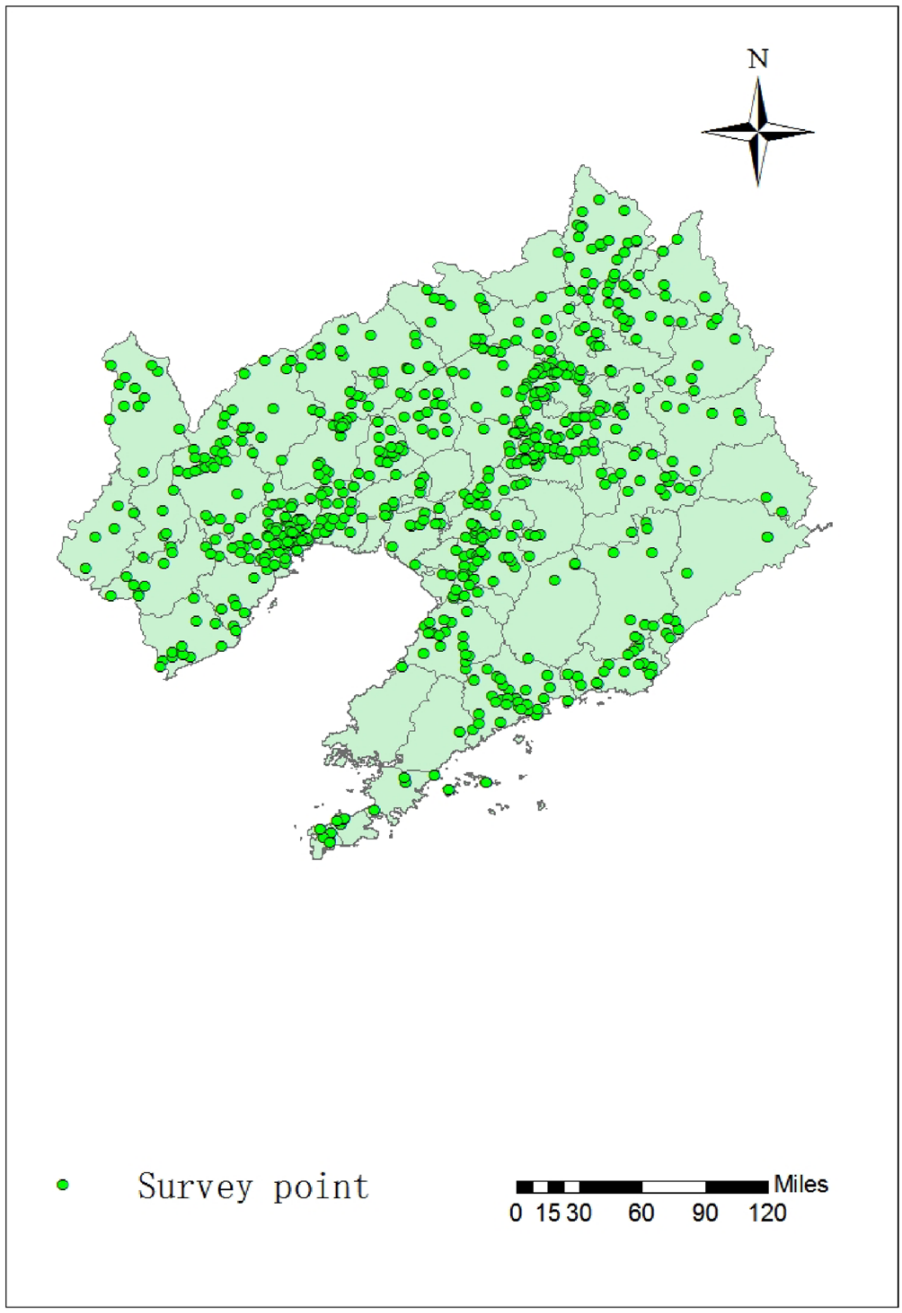
Distribution of sampling sites in Liaoning province, China. The map was created in ArcMap 10.2: https://www.esri.Com (Esri, California, USA).

710 survey points in agricultural regions were distributed in 14 cities of Liaoning province (Fig. 1). In general, the survey points were densely distributed in the Shenyang, Jinzhou, Huludao, Anshan and Tieling. The number of agricultural sites survey points was 399 in these cities. But obviously sparse in other cities. Benxi and Panjin had the least number of agricultural sites survey points at 22. On the one hand, the distribution pattern was consistent with the overall distribution of agricultural regions throughout Liaoning, and these points in the area with large planting regions. On the other hand, the obtained data of this study had a good representation, because it contained current common different cultivated land types in Liaoning. Due to the large proportion of food, fruit and vegetables in the diet, the main types of agriculture in the survey sites were food crops, fruit plantations, and vegetable plantations. In particular, the sampling sites of major crops such as corn and rice were more densely distributed. In addition, soil pH values of 710 survey sites are shown in Table 1, among which the soil pH values are less than 6.50, accounting for 63.95% of the total number of points. The activity of heavy metals in the soil under acidic conditions will increase compared with neutral and alkaline conditions^38^. Therefore, it should pay more attention to prevent soil from acidification.

### Overview of heavy metals concentration in soil

**Figure 2:**
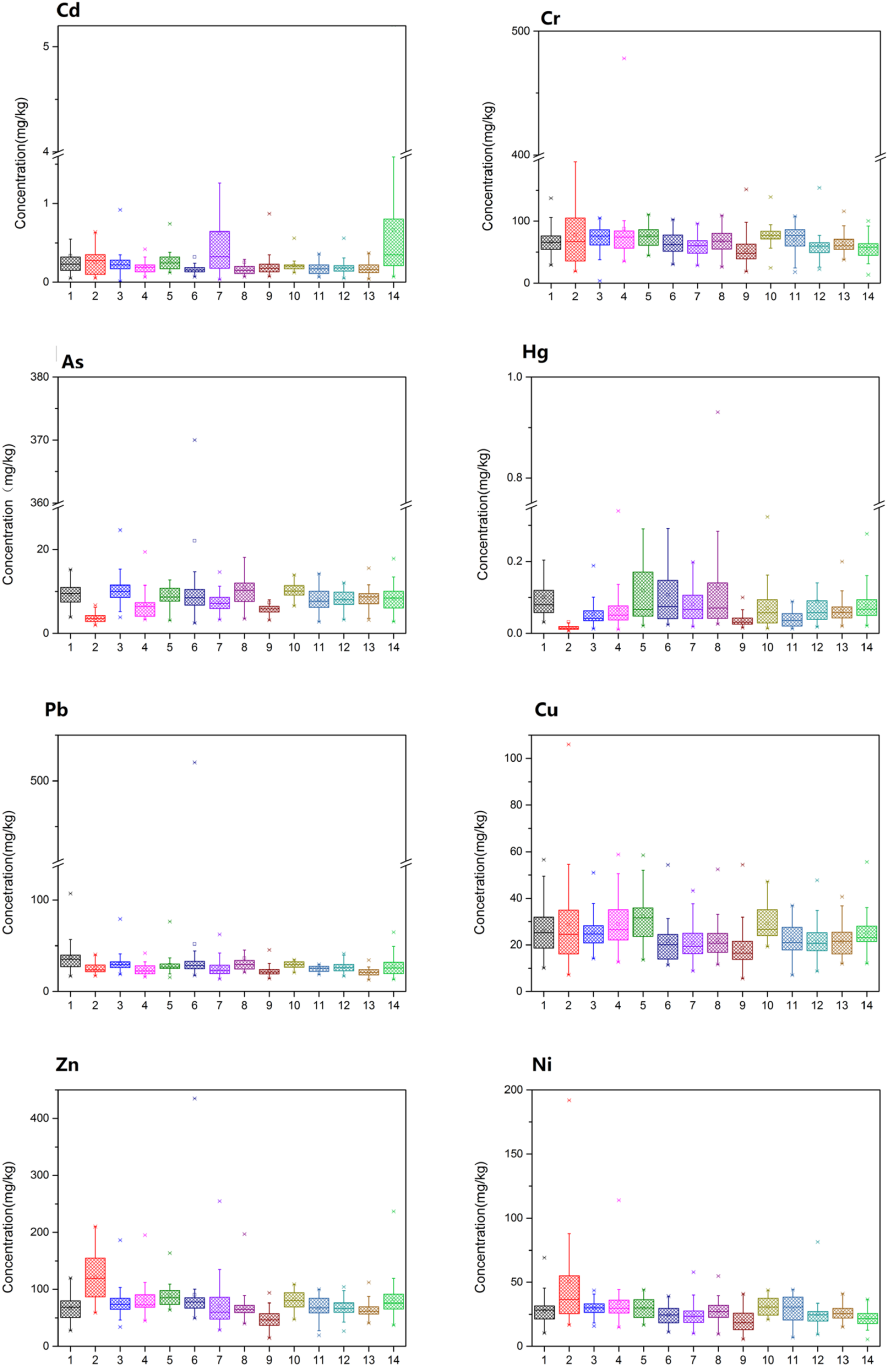
Concentrations of heavy metals in agricultural regions. The Box-and-Whisker plots show the minimum value(lower whisker), 25th quartile, median, 75th quartile, and maximum value (upper whisker) and outlier (·). (Code: 1: Shenyang, 2: Dalian, 3: Anshan, 4: Fushun, 5: Benxi, 6: Dandong, 7: Jinzhou, 8: Yingkou, 9: Fuxin, 10: Liaoyang, 11: Panjin, 12: Tieling, 13: Chaoyang, 14: Huludao).

The mean concentrations and percentile values of heavy metal are shown in Fig. 2. The mean concentrations of Cd (0.56 ± 0.78 mg/kg, 0.76 ± 0.86 mg/kg, 0.50 ± 0.16 mg/kg) were higher than the standard (GB15618-2018) in Jinzhou, Huludao and Benxi (0.30 mg/kg). In most cities, 75th and 95th percentile values of As, Cd, Cr, Pb, Cu and Ni exceeded the environmental quality standard. On the whole, the situation of Cd contaminated was not optimistic. The Cd 50th percentile values of Jinzhou and Huludao were already higher than the threshold value. The pollution situation in these two areas was relatively serious. It was similar to the research results of other scholars^39^. Except Panjin and Chaoyang, the Cd 75th and 95th percentile values of other cities exceeded the standard in varying degrees. Liaoning is an important area of the northeast industrial base, rich in mineral resources. The Cd concentration in the atmosphere around the industrial area is generally high, which enters the soil through rainfall or sedimentation, resulting in the higher Cd concentration in industrial soil^40^. The 95th percentile values of As and Pb in Dandong were far higher than the standard value. It was found that these points in Dandong are located around the Qingchengzi lead–zinc tailings, which further confirms that the mining industry contributes a lot to the heavy metal in the soil^41^. The 95th percentile values of other heavy metal content only slightly exceeded the standard in a few cities, but these were all within the controllable range. In general, the overall quality of farmland soil was still at low risk level except for several key areas.

### Geological accumulation assessment

**Figure 3:**
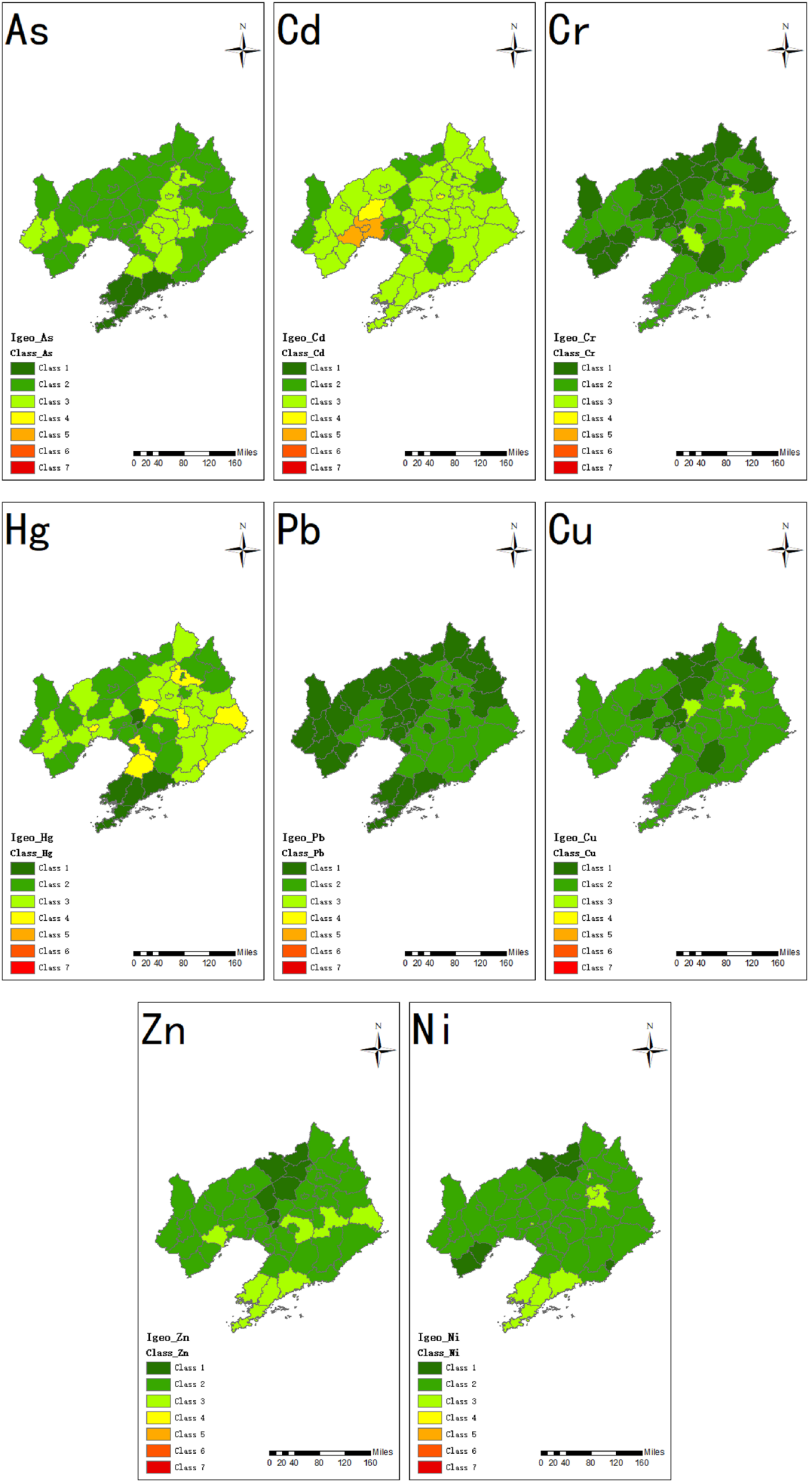
The geo-accumulation index of heavy metals in soil in Liaoning. The map was created in ArcMap 10.2: https:// www. esri. Com (Esri, California, USA). (Class 1: unpolluted; Class 2: unpolluted to moderately polluted; Class 3: moderately polluted; Class 4: moderately to heavily polluted; Class 5: heavily polluted; Class 6: heavily to extremely polluted; Class 7: extremely polluted).

Though the 14 cities were different in populations and sizes, the I_geo_ of 8 heavy metals in soil showed similar trends (Fig. 3). The I_geo_ values of As, Cr, Pb, Cu, Zn and Ni in most cities were at unpolluted or unpolluted to moderately polluted level (class 1, class 2). The order of mean I_geo_ values was Cd (1.53) > Hg (1.09) > As (0.69) > Zn (0.66) > Ni (0.58) > Cu (0.33) > Cr (0.06) > Pb (-0.01). The I_geo_ values for Cd showed the strongest variation, varying from unpolluted to heavily polluted level (class 5). The I_geo_ values for Hg in some cities (Benxi, Dandong, Chaoyang and Jinzhou) were at moderately to heavily polluted level. The I_geo_ values for Cd in Jinzhou and Huludao were at the heavily polluted level, and I_geo_ values of Cd in Yuhong (Shenyang) district was at the moderately to heavily polluted level. In general, the accumulation of heavy metals in soil showed an upward trend, which was mostly related to human activities.

### Potential ecological risk assessment

The order of potential ecological harm degree of heavy metal was as follows: Cd > Hg > As > Cu > Ni > Pb > Cr > Zn. The ER values for As, Cr, Hg, Pb, Cu, Zn, and Ni in all cities were in the low risk (class 1). The statistics of ER and RI for heavy metal are shown in Table S8. The ER values for Cd in 13 cities (except Jinzhou) were at low risk (Class 1), and the higher ER values for Cd were found in seven regions (Yuhong, Yixian, Taihe, Linghai, Lianshan, Longgang and Nanpiao). The RI values in 14 cities in Liaoning were lower than 150, indicating that these cities are facing lower potentially l ecological risk of heavy metal accumulation. In general, heavy metal in the topsoil of most cities in Liaoning had low or medium potential ecological risks.

### Human health risk assessment

**Figure 4:**
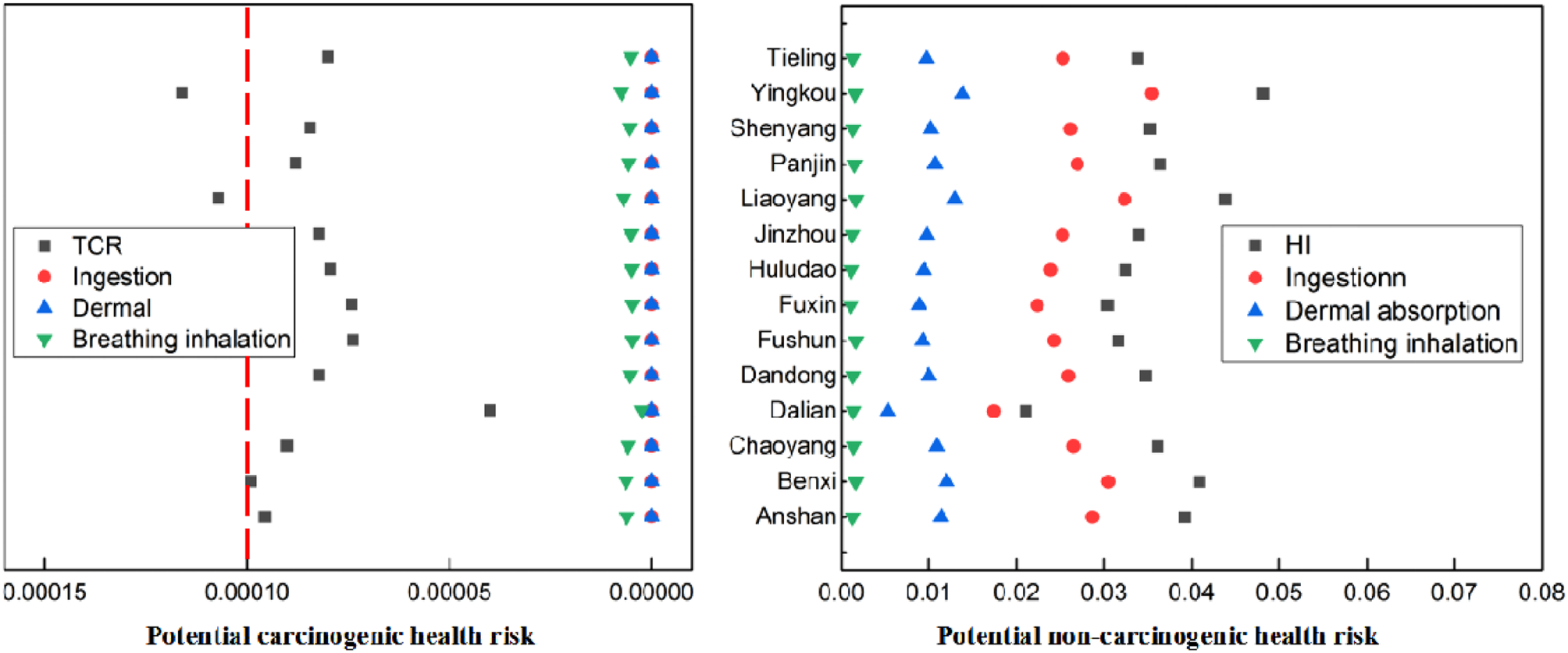
HI and TCR for adults caused by heavy metal in Liaoning province.

The HI and TCR values of adult citizens in Liaoning are shown in Fig. 4. The distributions of HQ for heavy metals in soil in the study region were ranked as follows: As > Pb > Cu > Cd > Zn > Ni > Cr > Hg. The HI < 1 indicates that the study area may have no non-carcinogenic risk for adults. However, the TCR values of heavy metals in some urban soil should be paid more attention. Because the other six heavy metals do not have carcinogenic slope factors, As and Cd were assessed for carcinogenic risk merely. The order of adult HQ values of three exposure routes was as follows: ingestion > dermal absorption > breathing inhalation. For adult, the oral ingestion of soil was the primary dangerous route of exposure. The TCR values of adult in Yingkou, Benxi, Huludao, Jinzhou and Anshan were higher than the threshold, while the TCR values of other cities were lower than the threshold. Anshan, Benxi, Jinzhou, Huludao and Yingkou were famous as the mineral mining around the country. Large quantities of heavy metals released in the environment through solid wastes, waste water, and waste gas which was discharged from the activities related to mineral mining and metal smelting^42,43^. This ultimately caused relatively high health risks from heavy metal exposure in these areas. As showed in Fig. 1, many survey points were distributed on the cultivated land around the mining area. The cultivated land around the mining area had unacceptable health risks.

### Comprehensive assessment of heavy metals pollution level

**Figure 5:**
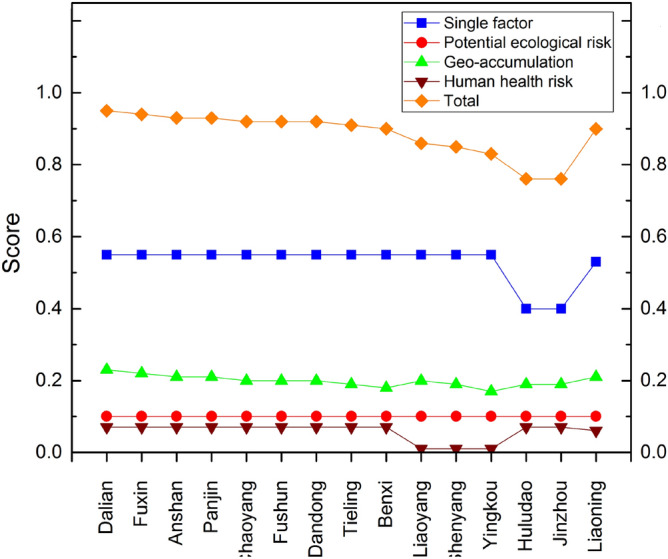
Comprehensive assessment of heavy metal pollution level.

In terms of the assessment method weight coefficient distribution, there was an obvious bias because the assessment with larger weight was concentrated in SF (0.55) and Geo (0.25). The weight ratio of the two factors was nearly 80%. The weight coefficient of PER and HHR was 0.10 respectively. This trend was consistent with the current practices of soil metal remediation, whose focus was metal content reduction in soil. In terms of heavy metal weight coefficient distribution, Cd (0.31) and Hg (0.41) accounted for more in the 8 heavy metals. The other six heavy metals (As, Cr, Pb, Cu, Zn, Ni) only accounted for 0.10, 0.02, 0.05, 0.05, 0.01, 0.05, respectively. The grading and assigning of the four evaluation methods are shown in Table 2. Comprehensive evaluation results of heavy metal pollution level are shown in Fig. 5. Total score of Liaoning pollution level was 0.8998. The score arrangement order of the evaluation method was similar to that weight coefficient order, M_SF_(0.5346) > M_GEO_(0.2067) > M_PER_(0.1000) > M_HHR_(0.0585). Generally, the pollution level of heavy metal in Liaoning was at low risk level, but it still needs to pay attention to the health risk of heavy metal and the input of heavy metal into the soil. The comprehensive assessment score of Dalian was 0.9536, ranking first among the 14 cities, indicating that the environmental quality is good. However, Huludao and Jinzhou comprehensive assessment score was 0.7594 respectively, ranked the last. It can clearly compare the pollution situation among different regions by comprehensive score.

**Table 2:** Comprehensive assessment scoring criteria.

Assessment method	Score	Scoring basis	Reference
Single factor assessment	1.00	Pi < 1	Xu et al.^28^ Yuan and Lei^52^
	0.70	Pi = 1	
	0.10	Pi > 1	
Geo-accumulation assessment	1.00	Igeo ≤ 0	Müller^31^ Ji et al.^32^
	0.86	0 < Igeo ≤ 1	
	0.71	1 < Igeo ≤ 2	
	0.57	2 < Igeo ≤ 3	
	0.43	3 < Igeo ≤ 4	
	0.29	4 < Igeo ≤ 5	
	0.14	Igeo > 5	
Potential ecological risk assessment	1.00	RI < 150	Hakanson^33^ Hu et al. (2020)
	0.70	150 ≤ RI < 300	
	0.40	300 ≤ RI < 600	
	0.10	RI ≥ 320	
Human health risk assessment	1.00	HI < 1 and TCR < 0.000001	Wang et al.^51^ Zhao et al.^37^
	0.70	HI = 1 or 0.0001 < TCR ≤ 0.000001	
	0.10	HI > 1 or TCR > 0.0001	

The calculation formula of single factor pollution index is as follows: Pi = Ci/S, Pi is the single factor index of pollutants. Ci is the measured concentration (mg/kg). S is the heavy metal risk screening value (mg/kg) (GB 15618-2018). See supplementary materials for other details.

### Source analysis of heavy metals pollution

**Figure 6:**
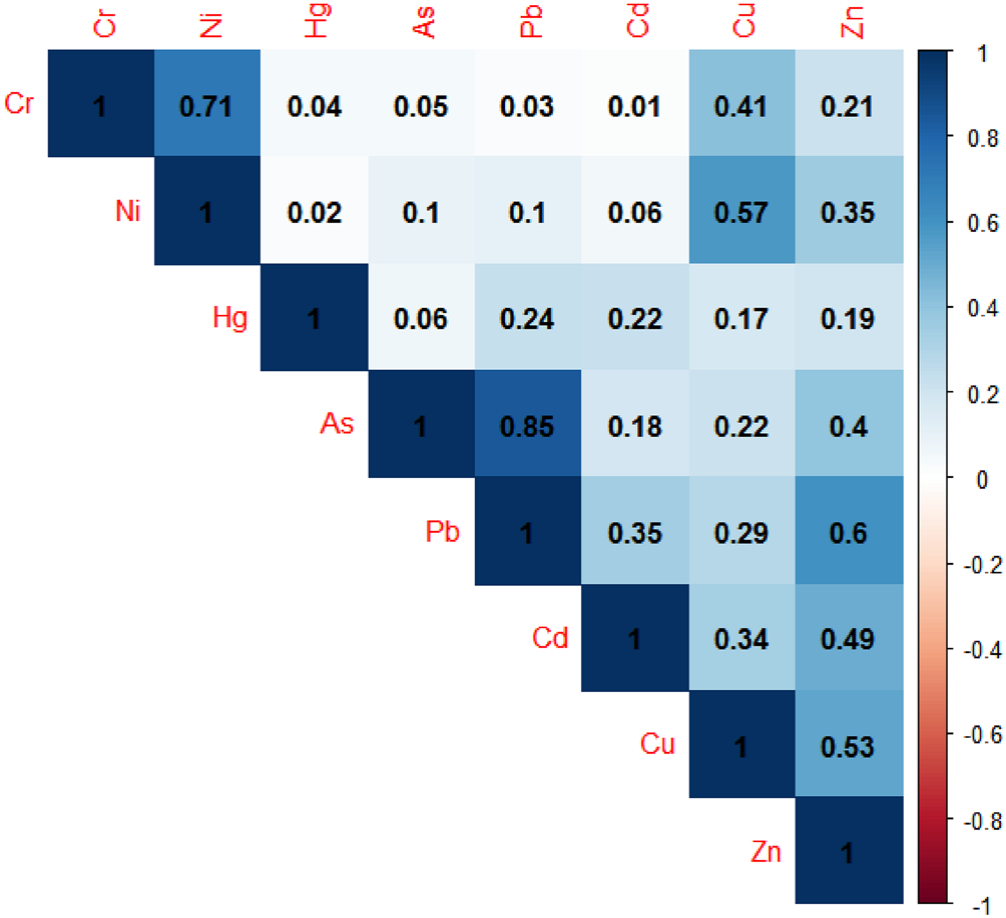
Correlation analysis of 8 heavy metals.

Heavy metals correlation analysis (CA) can judge the similarity of their sources. Generally, heavy metals with high correlation coefficient may have similar sources. Heavy metals with low correlation coefficient or negative correlation may have different sources. From the correlation analysis results (Fig. 6), it can be seen that there is a positive correlation between the eight heavy metals. It indicated that the pollution sources of the 8 heavy metals were similar. The correlation coefficients of As and Pb (r = 0.845, *P* < *0.01*), Cr and Ni (r = 0.713, *P* < *0.01*) were greater than 0.70, which indicated that the pollution sources of As and Pb, Cr and Ni may be the same. The correlation coefficients of heavy metals were less than 0.30, like As and Ni (r = 0.100, *P* < *0.01*), Hg and Cu (r = 0.174, *P* < *0.01*), indicating that these elements were not homologous. Other heavy metals showed moderate correlation, like As and Zn (r = 0.396, *P* < *0.01*), Cr and Cu (r = 0.411, *P* < *0.01*) indicating that their sources were complex.

Principal component analysis (PCA) of 8 heavy metals extracted three principal components. The cumulative variance contribution rate was 74.49%, which reflected the most of information about the sources of heavy metals in soil^44^. In principal component 1, the factor load values of Cu (0.734), Pb (0.749), As (0.628) and Zn (0.817) were large (Table 3). The pollution center values mostly appeared near non-ferrous metal smelts, which may be related to the industrial three wastes. In principal component 2, Cr (0.732), Cu (0.342) and Ni (0.708) were the main factors. According to the analysis of pollution characteristics, the areas with high pollution degree of Cr, Cu and Ni were mostly concentrated near metal products processing plants. This showed that heavy metals were discharged more in the process of metal products processing. In principal component 3, the factor load values of Cd (0.510) and Hg (0.638) were larger, and the areas with serious Cd pollution were mainly sewage irrigation areas. It reflected that sewage irrigation pollution was defined as the main pollution source.

**Table 3:** Principal component analysis of heavy metals in soil.

Heavy metals	Component		
	1	2	3
As	0.628	− 0.483	− 0.419
Cd	0.542	− 0.262	0.510
Cr	0.456	0.732	− 0.137
Hg	0.312	− 0.171	0.638
Pb	0.749	− 0.534	− 0.273
Cu	0.734	0.342	0.153
Zn	0.817	− 0.116	0.101
Ni	0.578	0.708	− 0.133

### Spatial analysis of pollution level

**Figure 7:**
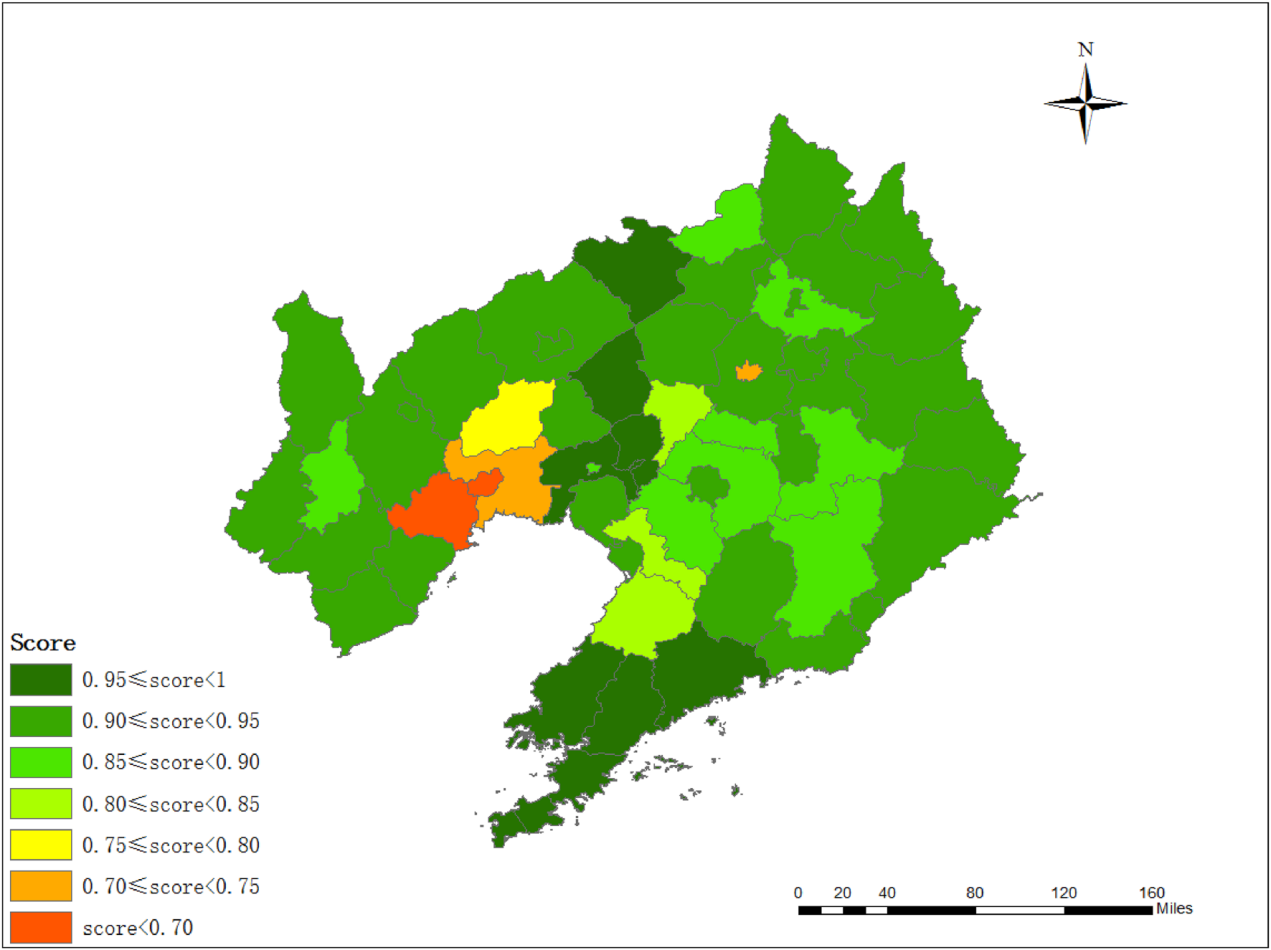
The spatial distribution map of the comprehensive assessment result. The map was created in ArcMap 10.2: https:// www. esri. Com (Esri, California, USA).

The spatial distribution map of the comprehensive evaluation score can understand the soil quality of the study area more directly (Fig. 7). The comprehensive results of pollution level also showed a certain feature. Soil pollution, centered on industrial and mining enterprises, spreads around and showed a downward trend. The pollution in the sewage irrigation area was distributed in bands. The spatial characteristics were caused by many factors such as soil parent material, agricultural activities and industrial activities^45,46^. Different cities have different degrees of heavy metal accumulation due to their own characteristics. Specific policies should be designed for different cities^47^. Such as Huludao, Jinzhou, Dandong and Benxi, their heavy metals pollution may be dominated by non-ferrous metal mining activities, and the government should resolutely close mines that do not meet environmental requirements^48^. In addition, enterprises should upgrade their production processes and strengthen existing environmental protection measures to reduce the total amount of heavy metal emissions. It is an effective measure to protect soil environmental quality to strengthen the standardized treatment of pollutants discharged by enterprises in cities with heavy metal industry^49^. The quantity and quality of fertilizer should be strictly controlled in cities where heavy metal accumulation is mainly agricultural. The accumulation of heavy metals in topsoil can cause vertical migration and aggravate groundwater pollution^50^. The accumulation of heavy metals in soil is a dynamic process which is affected by many factors^51^. A monitoring network should be established for long-term monitoring of dynamic changes in soil quality, which can provide accurate and up-to-date information for decision-maker^52^. It ensures that the carcinogenic and non-carcinogenic risks of heavy metal to the human body are controllable.

## Conclusions

The soil contamination assessment and investigation of soil quality have to be still regulated worldwide. This study has reviewed and presented a set of maps with comprehensive assessment score in Liaoning province. The current pollution level of heavy metals in soil and spatial features across Liaoning were demonstrated effectively by these maps. The concentrations of heavy metals and comprehensive assessment score in soil varied significantly. The result shows that Liaoning province assessment scores were 0.8998 (T), 0.9720 (SF), 1.0000 (PER), 0.8268 (GEO), and 0.5849 (HHR). The weight coefficients of 4 evaluation methods were 0.55(SF), 0.10 (PER), 0.25 (GEO), and 0.10 (HHR). 8 heavy metals weight coefficient (As, Cd, Cr, Hg, Pb, Cu, Zn, Ni) accounted for 0.10, 0.31, 0.02, 0.41, 0.05, 0.05, 0.01, 0.05, respectively. Heavy metal contents in most cities were greater than corresponding average background values. Most of the contaminated regions located in central and western Liaoning. Cd was found to be most polluted in soil in Liaoning. It needs to be vigilant about potential threat from Cd which was defined as mainly concerned pollution elements. Overall, the overall quality of farmland soil was still at low risk level except several key areas. These results can enhance our knowledge of heavy metal pollution status, potential health risks, and potential controlling factors for heavy metal accumulation in different regions across Liaoning. It also provides more comprehensive and up-to-date information for contributing to better soil management, soil remediation, and soil contamination control.

## Supplementary Information

Supplementary Information.

## Data Availability

The datasets used and/or analyzed during the current study are available from the corresponding author on reasonable request.
