# Supplementary material for: Comprehensive assessment of harmful heavy metals in contaminated soil in order to score pollution level
Source: Sci Rep. 2022 Mar 3;12:3552. doi: 10.1038/s41598-022-07602-9 (PMC8894455; doi:10.1038/s41598-022-07602-9)
Supplement: Supplementary file 1 — Supplementary Information. [file 41598_2022_7602_MOESM1_ESM.doc]

Comprehensive assessment of harmful heavy metals in contaminated soil in order to score pollution level

Haodong Zhao^1,2^, Yan Wu^1,2^, Xiping Lan^3^, Yuhong Yang^4^, Xiaonan Wu^1,2^, Liyu Du^1,2^

**Boxplot method:**

Boxplot provides a way to simply summarize a data set with only five points. These five points include midpoint, upper quartile (Q1), lower quartile (Q3), high and low of segment status. The boxplot is vividly divided into the whole range of center, extension and distribution state. Interquartile range (IQR) = upper quartile- lower quartile (IQR= Q3-Q1). Draw two line segments at Q3+1.5IQR and Q1-1.5IQR, which are the same as the median line. These two line segments are the outlier cutoff points, which are called the inner limit; Draw two line segments at Q3+3IQR and Q1-3IQR, which are called outer limits. The data represented by points outside the inner limit are all outliers. The outliers between the inner limit and the outer limit are mild outliers, and the outliers outside the outer limit are extreme outliers.

Table S1 Soil background mean value of Liaoning Province in 1982.

| Heavy metals | As | Cd | Cr | Hg | Pb | Cu | Zn | Ni |
| --- | --- | --- | --- | --- | --- | --- | --- | --- |
| Background concentration (mg·kg^-1^) | 3.00 | 0.05 | 40.60 | 0.02 | 18.00 | 12.00 | 30.00 | 12.00 |

Table S2 Seven classes comprising the geoaccumulation index.

| Class | Value | Soil quality |
| --- | --- | --- |
| 1 | Igeo≤ 0 | Practically uncontaminated |
| 2 | 0＜Igeo≤1 | Uncontaminated to moderately contaminated |
| 3 | 1＜Igeo≤2 | Moderately contaminated |
| 4 | 2＜Igeo≤3 | Moderately to heavily contaminated |
| 5 | 3＜Igeo≤4 | Heavily contaminated |
| 6 | 4＜Igeo≤5 | Heavily to extremely contaminated |
| 7 | Igeo＞5 | Extremely contaminated |

Table S3The relation between Er, RI and degree.

| Er | degree | RI | degree |
| --- | --- | --- | --- |
| Er＜40 | low risk | RI＜150 | low risk |
| 40≤Er＜80 | moderate risk | 150≤RI＜300 | moderate risk |
| 80≤Er＜160 | considerable risk | 300≤RI＜600 | considerable risk |
| 160≤Er＜320 | high risk | Er≥600 | high risk |
| Er≥320 | very high risk |  |  |

Table S4 Toxicity Coefficient of heavy metals.

| Heavy metal | As | Cd | Cr | Hg | Pb | Cu | Zn | Ni |
| --- | --- | --- | --- | --- | --- | --- | --- | --- |
| Tr | 10 | 30 | 20 | 40 | 5 | 5 | 1 | 5 |

Each column of comparison matrix A was normalized using Eq.(1). *n* represented the order of comparison matrix as well as the number of indicators.

_ii=_$bij/\sum\begin{matrix} n \\ i=1 \end{matrix}bij$ (1)

$Wi=\sum_{j=1}^{n} bij$ (2)

= [1,2,…,n] (3)

i= (4)

w=[w1,w2,…，w_i_]^T^, where *w* is the feature vector that is the weight value of each index. The maximum eigenvalue A max of comparison matrix using Eq. (5). *AW_i_* is the *i* element of the vector *AW*.

$\text{λmax}=\sum_{\text{i}=1}^{\text{n}} \text{（}AW\text{）}^{i}/nWi$ (5)

The consistency index (CI) was calculated using Eq. (6)

$CI=(\text{λmax-n)/(n-1)}$ (6)

The random consistency index (RI) (Table S7) was determined and the consistency ratio Cr calculated using Eq. (7).

$CR=CI/RI$ (7)

When CR < 0.1, the inconsistency of the comparison matrix is within the allowable range or there is no logical error in index comparison.

Table S5 Judgment matrix scale and meaning.

| b_ij_(Scaling) | Importance meaning |
| --- | --- |
| 1 | b_i_ is as important as b_j_ |
| 3 | b_i_ is slightly important than b_j_ |
| 5 | b_i_ is obviously important than b_j_ |
| 7 | b_i_ is strongly important than b_j_ |
| 9 | b_i_ is extremely important than b_j_ |

2,4,6,8 take the middle value of the above two adjacent judgments. The reciprocal of 1-9 numbers is opposite to the above meaning. For example, 1 / 5 means b_j_ is obviously more important than b_i_.

Table S6 Judgment matrix of importance for index.

| Layer | Index A | Index B | Index C | Index D |
| --- | --- | --- | --- | --- |
| Index A | 1 | A | b | c |
| Index B | 1/a | 1 | d | e |
| Index C | 1/b | 1/d | 1 | f |
| Index D | 1/c | 1/e | 1/f | 1 |

Table S7 Value of the random consistency indicator (RI).

| n | 1 | 2 | 3 | 4 | 5 | 6 | 7 | 8 | 9 | 10 | 11 |
| --- | --- | --- | --- | --- | --- | --- | --- | --- | --- | --- | --- |
| RI | 0.00 | 0.00 | 0.58 | 0.90 | 1.12 | 1.24 | 1.32 | 1.41 | 1.45 | 1.49 | 1.51 |

Table S8 The potential ecological risk index (ER) and integrated potential ecological risk index (RI) of soil heavy metals.

| City | As(Er) | Cd(Er) | Cr(Er) | Hg(Er) | Pb(Er) | Cu(Er) | Zn(Er) | Ni(Er) | RI |
| --- | --- | --- | --- | --- | --- | --- | --- | --- | --- |
| Shenyang | 1 | 1 | 1 | 1 | 1 | 1 | 1 | 1 | 1 |
| Dalian | 1 | 1 | 1 | 1 | 1 | 1 | 1 | 1 | 1 |
| Anshan | 1 | 1 | 1 | 1 | 1 | 1 | 1 | 1 | 1 |
| Fushun | 1 | 1 | 1 | 1 | 1 | 1 | 1 | 1 | 1 |
| Benxi | 1 | 1 | 1 | 1 | 1 | 1 | 1 | 1 | 1 |
| Dandong | 1 | 1 | 1 | 1 | 1 | 1 | 1 | 1 | 1 |
| Jinzhou | 1 | 2 | 1 | 1 | 1 | 1 | 1 | 1 | 1 |
| Yingkou | 1 | 1 | 1 | 1 | 1 | 1 | 1 | 1 | 1 |
| Fuxin | 1 | 1 | 1 | 1 | 1 | 1 | 1 | 1 | 1 |
| Liaoyang | 1 | 1 | 1 | 1 | 1 | 1 | 1 | 1 | 1 |
| Panjin | 1 | 1 | 1 | 1 | 1 | 1 | 1 | 1 | 1 |
| Tieling | 1 | 1 | 1 | 1 | 1 | 1 | 1 | 1 | 1 |
| Chaoyang | 1 | 1 | 1 | 1 | 1 | 1 | 1 | 1 | 1 |
| Huludao | 1 | 1 | 1 | 1 | 1 | 1 | 1 | 1 | 1 |

(Class 1: low risk; Class 2: moderate risk; Class 3: considerable risk; Class 4: high risk; Class 5: very high risk)

**Exposure assessment**

The general exposure equations used in this study are based on recommendations provided by several American and Canadian publications. To calculate levels of human exposure to heavy metals, the average daily intake (ADI) (mg/kg-day) equation by a given route is used:

$ADI=\frac{C\times IR\times EF\times ED}{BW\times AD}$ (8)

where C is the chemical concentration in a particular exposure medium (mg/L, mg/kg, mg/m^3^), IR is the ingestion rate (L/day, kg/day, m^3^/day), EF is the exposure frequency (day/per year), ED is the exposure duration (year), BW is the body weight of the exposed individual (kg), and AT is the time period over which the dose is averaged (day).

For heavy metals in contaminated soils, ingestion and dermal absorption play the most important roles among the potential exposure pathways. Considering these two pathways, the exposure dose was calculated using Eqs. (9), (10) and (11) adapted from the USEPA.

Ingestion:

(9)

where ADI_I_ is the average daily intake of heavy metals from soil ingestion (mg/kg-day), C_S_ is the heavy metal concentration found in the soil (mg/kg), and SIR is the ingestion rate of soil.

Dermal absorption:

(10)

where ADI_D_ is the average daily intake of heavy metals from dermal absorption (mg/kg-day), SA is the exposed skin surface area (cm^2^), AF is the adherence factor (mg/cm^2^-day), and ABS is the dermal absorption factor (unitless).

Breathing inhalation:

(11)

where ADI_B_ is the average daily intake of heavy metals from breathing inhalation (mg/kg-day), PM_10_ is the content of inhalable particles in the air (mg/m^3^), DAIR is the daily air breathing volume (m^3^/d), PIAF is the retention ratio of inhaled soil particles in vivo, FSPO is the proportion of particulate matter from soil in the air.

Non-carcinogenic risk assessment Non-carcinogenic hazards are typically characterized by the hazard quotient (HQ). The hazard quotient is defined as the quotient of the chronic daily intake, or the dose divided by the toxicity threshold value, which is referred to as the reference dose (RfD) of a specific chemical. The hazard quotient of a single chemical is determined by Eq. (12):

$HQ=\frac{ADI}{RfD}$ (12)

where RfD is the chronic reference dose for the chemical (mg/kg-day). To assess the overall potential for non-carcinogenic effects posed by more than one chemical, a Hazard Index (HI) approach has been applied. For a mixture of contamination, the hazard index of the mixture is calculated from Eq. (13):

$HI=\sum HQi=\sum\frac{ADIi}{RfDi}$ (13)

If the HI value is less than one, the exposed population is unlikely to experience obvious adverse health effects. If the HI value exceeds one, then adverse health effects may occur. Because no reference doses are presently available for directly evaluating dermal absorption exposure to contaminants, the USEPA has developed a method to extrapolate oral toxicity values for use in dermal risk assessment. RfD_ABS_ is calculated by using Eq. (14):

(14)

where RfD_ABS_ is the adjusted reference dose (mg/kg-day), RfD_o_ is the oral reference dose (mg/kg-day), and ABS_GI_ is the gastrointestinal absorption factor (unitless).

**Carcinogenic risk assessment**

Carcinogenic risks are estimated by calculating the incremental probability of an individual developing cancer over a lifetime as a result of exposure to the potential carcinogen. The slope factor (SF) converts the estimated daily intake of a toxin averaged over a lifetime of exposure directly to the incremental risk of an individual developing cancer:

$RISK=ADI\times SF$ (15)

where Risk is the unitless probability of an individual developing cancer over a lifetime and SF is the carcinogenicity slope factor (per mg/kg-day). Risks surpassing 1×10^−4^ are viewed as unacceptable, risks below 1×10^−6^ are not considered to pose significant health effects, and risks lying between 1×10^−4^ and 1×10^−6^ are generally considered an acceptable range, depending on the situation and circumstances of exposure. Similar with Rf_D_, according to the USEPA's extrapolation method, SF_ABS_ is calculated by using Eq. (16):

(16)

where SF_ABS_ is the adjusted slope factor (per mg/kg-day) and SF_O_ is the oral slope factor (per mg/kg-day).
